# Extraction of Antioxidant Compounds from Brazilian Green Propolis Using Ultrasound-Assisted Associated with Low- and High-Pressure Extraction Methods

**DOI:** 10.3390/molecules28052338

**Published:** 2023-03-03

**Authors:** Thiago Dantas Teixeira, Bruna Aparecida Souza Machado, Gabriele de Abreu Barreto, Jeancarlo Pereira dos Anjos, Ingrid Lessa Leal, Renata Quartieri Nascimento, Katharine Valéria Saraiva Hodel, Marcelo Andrés Umsza-Guez

**Affiliations:** 1 Department of Biotechnology, Health Science Institute, Federal University of Bahia, Salvador 40170-115, Brazil; 2 SENAI Institute of Innovation (ISI) in Health Advanced Systems (CIMATEC ISI SAS), Senai Cimatec University Center, Salvador 41650-010, Brazil; 3 Department of Food and Beverages, Applied Research Laboratory of Biotechnology and Food, Senai Cimatec University Center, Salvador 41650-010, Brazil; 4 Postgraduate Program in Biotechnology-Northeast Biotechnology Network (RENORBIO), Institute of Health Sciences, Federal University of Bahia, Salvador 40170-115, Brazil

**Keywords:** formononetin, *p*-coumaric acid, rutin, antioxidant capacity, alcoholic extraction, supercritical extraction, PCA

## Abstract

The demand for bee products has been growing, especially regarding their application in complementary medicine. *Apis mellifera* bees using *Baccharis dracunculifolia* D.C. (*Asteraceae*) as substrate produce green propolis. Among the examples of bioactivity of this matrix are antioxidant, antimicrobial, and antiviral actions. This work aimed to verify the impact of the experimental conditions applied in low- and high-pressure extractions of green propolis, using sonication (60 kHz) as pretreatment to determine the antioxidant profile in the extracts. Total flavonoid content (18.82 ± 1.15–50.47 ± 0.77 mgQE·g^−1^), total phenolic compounds (194.12 ± 3.40–439.05 ± 0.90 mgGAE·g^−1^) and antioxidant capacity by DPPH (33.86 ± 1.99–201.29 ± 0.31 µg·mL^−1^) of the twelve green propolis extracts were determined. By means of HPLC-DAD, it was possible to quantify nine of the fifteen compounds analyzed. The results highlighted formononetin (4.76 ± 0.16–14.80 ± 0.02 mg·g^−1^) and *p*-coumaric acid (<LQ—14.33 ± 0.01 mg·g^−1^) as majority compounds in the extracts. Based on the principal component analysis, it was possible to conclude that higher temperatures favored the release of antioxidant compounds; in contrast, they decreased the flavonoid content. Thus, the obtained results showed that samples pretreated with 50 °C associated with ultrasound displayed a better performance, which may support the elucidation of the use of these conditions.

## 1. Introduction

The demand for apiculture products has been growing, especially regarding their application in complementary medicine [1], as is the case of propolis. The composition of propolis is strongly associated with its botanical and geographic origin [2,3], but generally its centesimal composition is treated and described in a generic way [4]. *Apis mellifera* bees using *Baccharis dracunculifolia* D.C. (*Asteraceae*) as a substrate produce green propolis, classified by Park et al. [5] along with other types of Brazilian propolis. *B. dracunculifolia*, also widely known as “Alecrim do campo”, is native to the Southeast and South regions of Brazil and has been the subject of different investigations for ethnomedicinal, phytochemical and pharmacological purposes [6].

Over the years, records in the literature have been demonstrating the bioactive potential of this resin [7]. Among the examples of bioactivity of this matrix are the antioxidant, antimicrobial, anti-inflammatory, antiparasitic, antiviral, and antitumor actions [8,9,10,11]. In their studies, Silveira et al. [12] showed that the antiviral action of green propolis was promising in the treatment of patients with COVID-19, reducing hospitalization time and the development of kidney damage (a common sequela in patients with the disease). Silva-Beltrán et al. [13] verified the efficacy of the extract of green propolis as well as of some individual compounds present therein against human coronavirus 229E. Sokolonski et al. [14] showed the antifungal action of green propolis against *Candida albicans* isolates. The bioactivity of this matrix is mainly due to the presence of phenolic compounds (flavonoids, phenolic acids and their esters) in its chemical composition [15].

These bioactive compounds present in different types of propolis can be extracted using different methods and solvents [10], which can result in different chemical profiles of extracts [16]. Conventional methods for extraction of biocomposites, such as Soxhlet extraction, have some disadvantages, such as the high consumption of organic solvents, degradation of bioactive compounds by exposure to high temperatures, and the amount of time required to perform these techniques. On the other hand, the non-conventional methods are characterized by shorter operational time, low environmental impact, besides allowing the obtainment of extracts with greater purity [17,18].

Among the non-conventional methods, the extraction with supercritical fluid presents desirable characteristics in what concerns the extraction of thermosensitive compounds. Once it allows the use of low temperatures, providing a minor degradation of sample constituents, besides eliminating eventual problems with residual solvents [19,20,21], the suppression of the solvent/extract separation step becomes possible. These properties are of fundamental importance for the extraction of natural products, where the quality of the final product depends directly on the integrity of the biocomposites present in it [22,23].

Efforts have been made to improve conventional methods, applying alternatives that improve the yield and reduce the time and costs of the extraction step [24,25]. One alternative is the increasingly common use of sonication as a pretreatment in extraction processes of bioactive compounds in plant matrices [26,27]. The propagation of mechanical ultrasound waves provokes the phenomenon of acoustic cavitation in the sample, which induces a series of compressions and rarefactions in the solvent molecules, leading to bubble formation on the solute surface [28]. These bubbles implode, generating an increased interaction between solute and solvent due to the increased penetrability through the open channels on the sample surface [29].

In this context, the present work aimed to verify the impact of different experimental conditions applied to low-pressure (ethanolic) and high-pressure (supercritical) extractions of green propolis, using sonication (60 kHz) as pretreatment, as well as to determine the profile of phenolic compounds in the extracts obtained.

## 2. Results and Discussion

### 2.1. Antioxidant Profile of Green Propolis Extracts (Total Phenolic Compounds, Flavonoid Content and Antioxidant Capacity)

**Figure 1:**
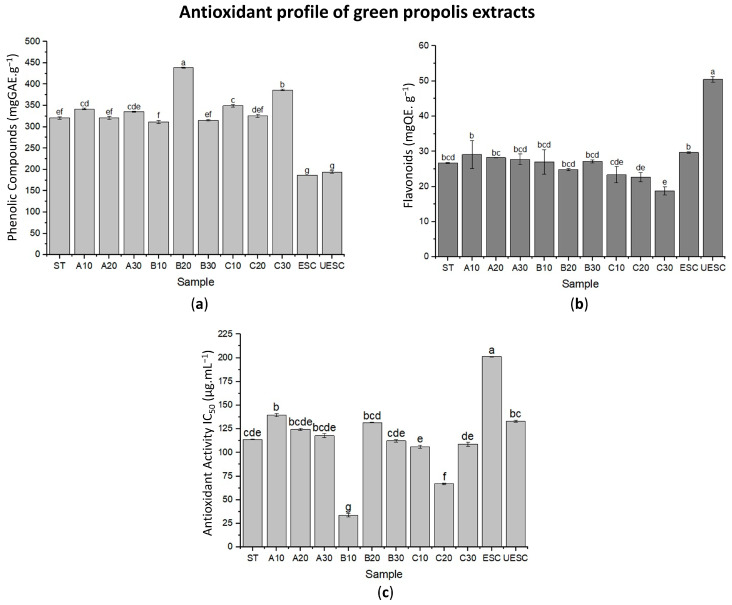
Antioxidant profile of green propolis extracts: (**a**) Determination of total phenolic compounds (mgGAE·g^−1^); (**b**) flavonoids (mgQE·g^−1^) and (**c**) antioxidant capacity to DPPH (IC_50_) of different green propolis extracts obtained by ethanolic (LPE) and supercritical extraction (SFE). Error bars represent the standard deviation (*n* = 3). Values presenting the same letter do not show significant differences (*p* > 0.05) in Tukey’s test at 95% confidence.

Propolis is the third most important component of bee products. This product is highly rich in bioactive compounds such as phenolic compounds, esters, flavonoids, terpenes, among other important organic compounds [1]. Figure 1 presents the results for the content of total phenolic compounds (TPC), flavonoids (FT) and antioxidant capacity (AC) of the extracts of different green propolis samples obtained by the two extraction methods as per Figure 1c (conventional ethanolic/low pressure (LPE) and supercritical/high pressure (SFE)). In general, a significant variation (*p* < 0.05) was observed for TPC, FT and CA among the green propolis extracts obtained by different methods. TPC showed a variation of 57.5% among the extracts (186.81 ± 0.32 to 439.05 ± 0.90 mgGAE·g^−1^, ESC samples and B20, respectively) (Figure 1a), while FT varied by 63% (18.82 ± 1.15 to 50.47 ± 0.77 mgQE·g^−1^, samples C30 and UESC, respectively) (Figure 1b), whereas AC (IC_50_) varied by 83% (33.86 ± 1.99 to 201.29 ± 0.31 µg.mL^−1^, samples B10 and ESC, respectively. The mean and standard deviation of the values obtained in each of the analyses are shown in Table S1 (Supplementary Materials).

Importantly, the extracts obtained by LPE showed a 29% variation in TPC (311.74 ± 3.43 to 439.05 ± 0.90 mgGAE·g^−1^, samples B10 and B20, respectively) while those obtained by SFE varied by approximately 4% (186.81 ± 0.32 to 194.12 ± 3.40 mgGAE·g^−1^, samples ESC and UESC, respectively). Therefore, on average, LPE extracts showed 45% higher TPC than SFE. Since the low polarity of supercritical CO_2_ provides it with a high power to solubilize compounds with similar polarity, such as waxes, this may explain the lower TPC yield in the extracts obtained by SFE. Waxes are poorly soluble in ethanol, which contributes to the increased interaction of this solvent with the phenolic compounds present in the propolis sample, increasing the yield in this type of extraction. In contrast, CO_2_ displays weaker interaction with the phenolic compounds, leading to a decrease in the extraction yield. Although solvent polarity is an important parameter, this is not the only preponderant characteristic in the extraction process, since aspects such as solute/solvent interaction are of equal importance [30]. In their studies using *B. dracunculifolia*, Casagrande et al. [31] evidenced that the extracted phenolic compounds are strongly influenced by the concentration of ethanol (40%, 60% and 80%) used in the extraction solution. Devequi-Nunes et al. [8] evidenced that the brown, green and red propolis extracts obtained by LPE showed higher TPC content than their respective extracts obtained by SFE, and the values of both green propolis extracts corroborate those obtained in the present study (374.10 and 174.31 mgGAE·g^−1^, respectively).

The UESC sample (60 KHz, 50 °C and 20 min) showed a TPC value (194.12 ± 3.40 mgGAE·g^−1^) of approximately 4% higher than the ESC (186.81 ± 0.32 mgGAE·g^−1^). Of the LPE extracts, the sample with the highest TPC was B20 (439.05 ± 0.90 mgGAE·g^−1^), pretreated with ultrasound under the same conditions as the UESC extract, and this was 27% higher than the ST sample (320.97 ± 3.07 mgGAE·g^−1^). De Souza et al. [25] detected a similar effect in obtaining CFT in grape seed oil, where samples pretreated with ultrasound obtained higher TPC concentrations. This effect can be explained by the rupture of the material due to acoustic cavitation caused by sonication, forming pores in its structure [32]. Due to the pores opened by acoustic cavitation, phenolic compounds are easily released from the matrix [26]. In their studies, Taddeo et al. [33] obtained a 28% increase in the yield of biocompounds in Italian propolis extracts using conventional solvent extraction combined with ultrasound exposure which corresponds with the yield reported in the present study for samples obtained by LPE.

Regarding the FT, a different behavior was observed, where the LPE samples obtained 36% lower yield (between 18.82 ± 1.15 and 29.14 ± 3.98 mgQE·g^−1^) when compared to the SFE extracts (between 29.68 ± 0.26 and 50.47 ± 0.77 mgQE·g^−1^). Among the samples obtained by LPE, the one that obtained the highest FT concentration was A10 (60 KHz, 25 °C and 10 min) (29.14 ± 3.98 mgQE·g^−1^). In contrast, sample C30 (60 KHz, 75 °C and 30 min) (18.82 ± 1.15 mgQE·g^−1^) obtained 35% lower yield and was pretreated at higher temperature and treatment time conditions, which can be explained by the thermosensitivity of flavonoids [34]. Since sample C30 had three times the temperature and exposure time conditions of sample A10, it is possible that degradation of flavonoid molecules occurred in the sample. In a study by Liu, Wang and Cai [35], it was shown that the extraction of flavonoids in *Scutellaria baicalensis* (Chinese medicinal plant) had decreased yield in extractions conducted with temperature higher than 60 °C due to loss of activity and degradation of flavonoids.

The UESC sample presented flavonoid concentration 1.7 times higher than its control (ESC). The authors De Andrade et al. [24] indicate that the pretreatment of grape skin samples with ultrasound improves the yield of FT. This can be justified because the ultrasound waves stimulate the formation of small bubbles subjected to rapid compression and expansion, causing rapid local increase in temperature and pressure, which facilitates the solubilization of compounds present in the matrix [26]. In contrast, sample B20, pretreated under the same conditions as UESC (60 KHz, 50 °C and 20 min), was 50.8% lower than this sample obtained by SFE considering the concentration of FT (Figure 1b). Saito et al. [36] observed the same pattern in the amount of flavonoids, where supercritical extracts of red propolis showed these constituents in larger amounts when compared to ethanolic extracts. Similar behavior was also reported by Martinez-Correa et al. [37], who observed higher amount of flavonoids in supercritical extract of *Eugenia uniflora* when compared to ethanolic and aqueous extracts of this matrix. This fact indicates that supercritical extraction has greater selectivity in obtaining flavonoids.

The research for natural matrices with high content of compounds with antioxidant capacity has increased considerably in recent years, especially due to the potential benefits that these components present considering biological environments [38,39]. Within this perspective, it was observed that the IC_50_ value was lower in ethanolic sample B10 (60 KHz, 50 °C and 10 min) (33.86 ± 1.99 µg.mL^−1^). This value was approximately 2.8 times lower than the extract with the highest antioxidant capacity reported by Zhang et al. [40] for Brazilian green propolis (93.51 µg.mL^−1^). Considering that, the IC_50_ expresses the sample concentration required to neutralize the DPPH radical by 50%; the lower this value, the higher the antioxidant potential of the sample. Within this perspective, the sample with the lowest antioxidant capacity, represented by the highest IC_50_ value, was the supercritical ESC (201.29 ± 0.31 µg.mL^−1^). These data can be explained since the TPC of the B10 extract is approximately 1.6 times higher than that of the ESC sample. This indicates that the phenolic compounds comprising this pretreated sample, or even the presence of other compounds released in the pretreatment, contribute to the antioxidant potential [41].

Additionally, in the samples obtained by SFE, it was possible to notice that the UESC sample had higher AC (IC_50_: 133.17 ± 1.09 µg.mL^−1^) when compared to its ESC control (IC50: 201.29 ± 0.31 µg.mL^−1^). This effect is consistent with the FT concentration of these samples, since in the pretreated sample the concentration of these compounds was approximately two times higher than that in the control sample. Flavonoids are biocompounds that are oxidized by free radicals, resulting in a more stable and less reactive radical, providing these compounds with the antioxidant potential [42].

Considering the potential application of samples with higher antioxidant capacity, such as sample B10, there is a great need for the use of matrices with these properties in scientific and technological studies. For example, it has been shown that the presence of antioxidant components in propolis samples is related to the increase in its anti-aging [43], anti-inflammatory [44], and anti-tumor activities [45], among others. From the technological development point of view, the presence of propolis extracts with antioxidant properties in food packaging has contributed to the increase in the shelf life of products [46,47,48]. This perspective reinforces the need for studies such as this one, which have, among their objectives, the aim of elucidation of the antioxidant property of natural matrices.

**Figure 2:**
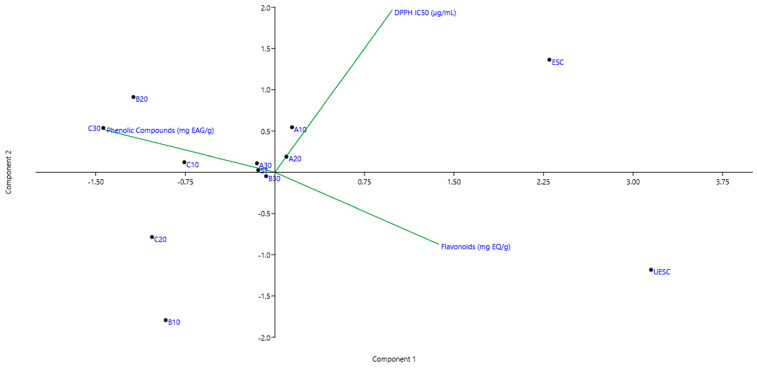
Principal component analysis of the samples obtained by the different extraction methods (PC scores) according to the number of loadings (content of phenolic compounds, flavonoids, and DPPH radical scavenging ability).

Figure 2 presents the correlation of extraction methods with respect to the content of phenolic compounds (mgGAE·g^−1^), flavonoids (mgQE·g^−1^) and DPPH radical scavenging capacity (IC_50_, µg.mL^−1^) through Principal Component Analysis (PCA) in order to detect the principal component that best describes the highlighted influences of this study. The principal components (PC1 *x*-axis: 64.34% of score value and PC2 *y*-axis: 25.95% of score value, corresponding to 90.29% of total cumulative variance) differentiate the extraction methods according to TPC, FT and AC properties. Figures S1 and S2 show the graph of loadings PC1 and PC2, respectively.

Treatments such as A10 and A20 were grouped in the right quadrant (positive) along with the DPPH variable (Figure 2), indicating that samples with lower temperature and time of ultrasound exposure had lower antioxidant capacity due to the high IC_50_ value. The ESC treatment, despite using higher temperature and time, was also grouped in the same quadrant due to its high IC_50_ value; however, the lower phenolic content of this sample explains its low antioxidant capacity. Inversely located on the left side (negative), samples B10 and C20 were grouped together (Figure 2), indicating that the increase in temperature may be directly related to favoring the extraction of compounds with antioxidant potential, since these samples had the lowest IC_50_ values. On the right side (negative) the UESC treatment was grouped along with the flavonoids variable (Figure 2). This may indicate that the supercritical extraction associated with ultrasound exposure directly contributed to flavonoid extraction due to the high concentration obtained in this extract (Figure 1b).

Conversely, on the opposite left side (positive and negative) are samples C10, C20 and C30, that, despite sonication, showed the lowest flavonoid contents (Figure 1b). This shows a trend of higher temperatures associated with a longer exposure time being able to affect flavonoid extraction. Observing the left side (upper), it was noted that the B20 treatment was the one that best represented the variable TPC along with C10 and C30 (Figure 2), since they obtained higher concentrations of these compounds (Figure 1a). On the right side (positive and negative) the ESC and UESC treatments are positioned. From this, it is possible to note a tendency that ethanol extraction associated with sonication favors the extraction of phenolic compounds.

### 2.2. Quantification of Compounds by HPLC

Among the fifteen compounds analyzed in the different extracts, it was possible to identify and quantify nine compounds in most samples. Among them are formononetin (from 4.76 ± 0.16 to 14.80 ± 0.02 mg·g^−1^); *p*-coumaric acid (<LQ—14.33 ± 0.01 mg·g^−1^); quercetin (from 0.67 ± 0.02 to 2.45 ± 0.00 mg·g^−1^); gallic acid (<LQ—2.78 ± 0.01 mg·g^−1^); kaempferol (from 0.34 ± 0.01 to 2.51 ± 0.03 mg·g^−1^); caffeic acid (0.19—3.02 ± 0.30 mg·g^−1^); catechin (from 0.52 ± 0.00 to 1.37 ± 0.03 mg·g^−1^); epicatechin (from 0.22 ± 0.03 to 0.98 ± 0.01 mg·g^−1^) and rutin (from 0.15 ± 0.01 to 10.00 ± 0.03 mg·g^−1^) (Table 1). Determining and quantifying the bioactive compounds of propolis is of great importance, since each type of propolis has unique characteristics, and when its main components are determined, the type of propolis can be targeted for specific therapeutic indications [49].

In most samples obtained by LPE, the majority compounds were formononetin (from 4.76 ± 0.16 to 14.80 ± 0.02 mg·g^−1^) and *p*-coumaric acid (<LQ—14.33 ± 0.01 mg·g^−1^) (Table 1). Formononetin is an isoflavone commonly found in red propolis samples that has been reported in the literature for its fungicidal, antioxidant, gastroprotective, and dyslipidemic regulating properties [50,51,52]. Despite being considered as a biomarker of red propolis, formonononetin was reported as a majority compound in most extracts obtained from green propolis, indicating the possibility that bees of the *Apis mellifera* species also occasionally collect plants containing this biocompound [53]. The presence of formononetin has also been reported in samples of green propolis from the state of Minas Gerais, located in southeastern Brazil [54]. These findings reinforce the idea of the chemical complexity of propolis, which has encouraged the publication of studies aimed at its quality control and standardization [55]. *p*-Coumaric acid and its prenylated derivatives are phenolic acids widely known as biomarkers of Brazilian green propolis [56,57]. Ferreira et al. [58] showed that this biocompound can act directly and indirectly in mitigating inflammatory processes. Celińska-Janowicz et al. [59] showed that *p*-coumaric acid has the ability to cause apoptosis in tongue squamous cell carcinoma cells (CAL-27). Many of these effects are directly associated with its AC [60].

Among the extracts obtained by LPE, those with the highest AC were B10 (IC_50_: 33.86 ± 1.99 µg.mL^−1^) and C20 (IC_50_: 67.1 ± 0.86 µg.mL^−1^). From the results of the chromatographic analyses, it was possible to note the high concentration of formononetin and *p*-coumaric acid simultaneously in these samples, which may have directly influenced the ability to neutralize the DPPH free radical. In their in vitro study to determine the antioxidant potential of formononetin, Vishnuvathan et al. [61] concluded that the ability of this substance to neutralize the DPPH radical increased in a concentration-dependent relationship. Shen et al. [62] detected a similar relationship in an in vitro study of the antioxidant potential of *p*-coumaric acid against the DPPH radical.

In the samples obtained by SFE, it was possible to observe that the ESC sample presented formononetin (8.39 ± 0.03 mg·g^−1^) and *p*-coumaric acid (7.87 ± 0.03 mg·g^−1^) as the majority compounds. In contrast, the UESC sample showed lower levels of these compounds (4.76 ± 0.16 mg·g^−1^ and <LQ, respectively), but the high amount of rutin (10.00 ± 0.03 mg·g^−1^) may have directly interfered with the antioxidant potential of this sample (Table 2). Selvaraj et al. [63] concluded that rutin has antioxidant capacity against both DPPH and ABTS [2,2′-azino-bis (3-ethylbenzothiazolin) 6-sulfonic acid] radicals, the antioxidant potential of which grows in a concentration-dependent relationship. In a study by Silva-Beltrán et al. [13], it was shown that some of the phenolic compounds present in green propolis have antiviral properties against human coronavirus (HCoV 229-E), where quercetin reduced the cytopathogenicity of the virus by up to 90%, followed by caffeic acid (80–90%) and rutin (75%).

**Figure 3:**
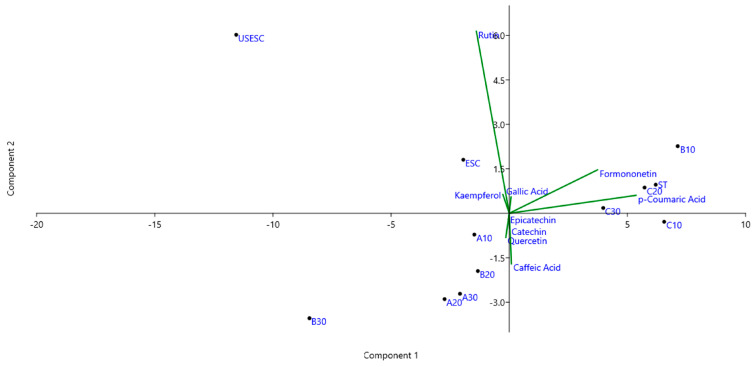
Principal component analysis of the samples obtained by different extraction methods according to the amount of phenolic compounds by HPLC.

The PCA presented in Figure 3 was performed in order to evaluate the influence of the extraction methods with respect to the content of the nine phenolic compounds quantified by HPLC (Table 1). The first two components (PC1 and PC2) explained 94.30% of the data, demonstrating that the influence of the extraction methods on the concentration of these compounds is strong. PC1 *x*-axis had the highest score value (89.59%), while PC2 *y*-axis had the lowest score value (4.71%). Observing the right side (positive), it is possible to notice the grouping of treatments B10, C10, C20 and C30 together with the variable formononetin and *p*-coumaric acid. On the other hand, on the left side (negative) are opposed treatments such as A10, A20 and A30 that have lower content of these biocompounds, which may be an indication that higher temperatures favored the extraction of these phenolic compounds.

Both the ESC and UESC samples were positioned to the left-positive side, showing that it is possible that supercritical extraction and/or pretreatment will have disfavored the extraction of the caffeic acid compound. However, the samples obtained by SFE occupied this position along with the variable rutin due to the higher concentration obtained for this compound. It is possible, analyzing the control sample (ESC), that the pretreatment with ultrasound may have been responsible for the release of kaempferol and rutin in the UESC sample. Since most of the samples obtained by LPE are opposed to the rutin variable, it may be an indication that SFE may have been more selective for obtaining this compound.

## 3. Materials and Methods

### 3.1. Reagents

Ethanol (HPLC grade) and acetic acid (HPLC grade) were purchased from Merck Co. (Darmstadt, Germany), methanol (HPLC grade) and DMSO (dimethyl sulfoxide) were purchased from Sigma-Aldrich Chemical Co. (St. Louis, MO, USA). Cellulose ester membrane filters of 0.45 μm (SLCR025NS, Millipore 1Co. Bedford, MA, USA) were used. The carbon dioxide (CO_2_) used in the extraction presented 99.9% purity (White Martins Gases Industrials—São Paulo, Brazil). The gallic acid (CAS number 149-91-7), catechin (CAS number 7295-85-4), epicatechin (CAS number 490-46-0), trans-cinnamic acid (CAS number 140-10-3), narigenin (CAS number 67604-48-2), caffeic acid (CAS number 331-39-5), *p*-coumaric acid (CAS number 501-98-4), resveratrol (CAS number 501-36-0), formononetin (CAS number 485-72-3), rutin hydrate (CAS number 207671-50-9), quercetin (CAS number 117-39-5), kaempferol (CAS number 520-18-3), and myricetin (CAS number 529-44- 2) were purchased from Sigma-Aldrich Chemical Co. (St. Louis, MO, USA), and trans-ferulic acid (CAS number 537-98-4) was purchased from Fluka (Buchs, Switzerland). 

### 3.2. Obtaining Extracts from Green Propolis

The sample of green propolis used in this study was acquired from an apiary in the city of Carmo da Mata (Minas Gerais, Brazil). The sample was processed in a mill (Cadence, Santa Catarina, Brazil) to obtain a diameter between 52 and 92 μm, thus facilitating the extraction process and the uniformity of the material. The sample was kept at −10 °C in inert atmosphere conditions (N2) in a fractional form to avoid the oxidation of the material.

#### 3.2.1. Low Pressure Extraction (LPE)

Propolis extracts were prepared from the homogenization of 2 g of raw green propolis in 15 mL of ethanol (80%). The system was pretreated in an ultrasonic bath at a frequency of 60 KHz, varying the temperature conditions (A-25 °C, B-50 °C and C-75 °C) and exposure time (A, B, C—10, 20 and 30 min, respectively) (Table 2). Then, the system was stored in the dark for 7 days, with manual shaking for 5 min every 24 h. The extract was recovered by centrifugation (SIGMA Centrifuge 2–16 KL, USA) at 5000 rpm (10 °C) for 11 min, and the supernatant was transferred to glass test tubes (15 × 160 mm) and kept at 45 °C until complete evaporation of the solvent. A control (ST sample) was obtained, without ultrasonic bath pretreatment. All extracts were kept at 5 °C until use [64].

#### 3.2.2. Supercritical Fluid Extraction (SFE)

To obtain propolis extracts by supercritical extraction, an SFT-110 Supercritical Fluid Extractor (Supercritical Fluid Technologies, Inc., Newark, NJ, USA) was used. In each experiment, the extraction cell was composed of 5 g of ground green propolis with 5.5 mL of ethanol (80%) as co-solvent, as well as wool and glass beads. The system was pretreated in an ultrasonic bath at the frequency of 60 KHz, 50 °C for 20 min, since this treatment condition obtained higher TPC yield in the extracts obtained by LPE (Figure 1). The extraction conditions were the following: pressure—350 bar; temperature—50 °C; CO_2_ flow—6 g.min^−1^. The extraction time was approximately 2.5 h. The extracts, collected in glass vials, were stored under the shelter of light, in inert atmospheric conditions (N_2_) to avoid degradation of the constituents. A control (ESC sample) was obtained without pretreatment in an ultrasonic bath. The extracts were kept at 5 °C until the time of use [64].

### 3.3. Determination of Total Phenolic Compounds

The analyses for determination of total phenolic compounds in green propolis extracts were performed by the Folin–Ciocalteu’s spectrophotometric method using gallic acid as standard [65]. Ethanol 80% was used to solubilize the extracts to obtain a concentration of 20 mg.mL^−1^. Then, 0.5 mL of the extract solution was withdrawn and mixed with 2.5 mL of aqueous Folin–Ciocalteu solution (10%) and 2.0 mL of 7.5% sodium carbonate. The solution was placed in a thermoregulated bath at 50 °C for 5 min and then the absorbance was measured in a spectrophotometer (Lambda 25 UV/vis Systems—PerkinElmer, Washington, DC, USA) at 765 nm. The results of the concentrations of total phenolic compounds were compared with an analytical curve of gallic acid (mgGAE·g^−1^) (y = 0.0104x + 0.0688; R2 = 0.9976) under the same conditions. All analyses were performed in triplicate and expressed as gallic acid equivalents (mgGAE·g^−1^).

### 3.4. Determination of Flavonoid Content

The determination of the total flavonoid content of the green propolis extracts was performed in a spectrophotometer (Lambda 25 UV/vis Systems—PerkinElmer, Washington, DC, USA) at 415 nm. A solution with the propolis extracts was prepared using 2.0% aluminum chloride in methanol in 1:1 (*v*/*v*) solution [66]. The same procedure was performed using known solutions of quercetin standard to prepare an analytical curve (y = 0.0311x + 0.0259; R2 = 0.9987). In addition, a blank sample was prepared under the same conditions and the amount of flavonoids was expressed as quercetin equivalents (QE) (mgQE·g^−1^). All analyses were performed in triplicate.

### 3.5. Determination of the Antioxidant Capacity: DPPH Method

The evaluation of the antioxidant capacity of the extracts was performed with 1,1-diphenyl-2-picrylhydrazyl (DPPH), according to the methodology described by Brand-Williams et al. [67]. The green propolis extracts were diluted to five concentrations in triplicate. Then, 1.0 mL of each dilution was transferred to a test tube containing 3.0 mL of ethanolic DPPH solution (0.004%). After 30 min of incubation in the dark and at room temperature, the reduction in the DPPH free radical was measured by reading the absorbance in a spectrophotometer (Lambda 25 UV/vis Systems—PerkinElmer, Washington, DC, USA) at 517 nm. A blank sample was prepared using ethanol instead of the sample. The IC_50_ value (concentration in µg.mL^−1^ required of the extract to sequester 50% of the DPPH radical) was calculated using the straight line equation obtained by constructing the curve based on the concentrations of the extracts (Table S2).

### 3.6. Chromatographic Analysis of Green Propolis Extracts

The phenolic compounds (gallic acid, catechin, epicatechin, trans-cinnamic acid, naringenin, caffeic acid, *p*-coumaric acid, resveratrol, formononetin, rutin hydrate, quercetin, kaempferol, myricetin, O-dianiside, and trans-ferulic acid) were analyzed from the green propolis extracts. First, 10 mg of green propolis extracts obtained from the different extraction methods were prepared and dissolved in ethanol (HPLC grade). A 0.45 μm cellulose ester membrane filter (Micropore) was used to filter the samples, before injection into the chromatographic system. The chromatographic analyses were performed using a High-Performance Liquid Chromatography (HPLC) equipment (Shimadzu, Tokyo, Japan), composed of a pump (LC-20AT), supplied with an automatic injector (SIL-20AHT), degasser (DGU-205), diode array detector (DAD) (SPD-M20A) and a column oven (CTO-20A). The method used to promote chromatographic separation was adapted from Daugsch (2007) and Machado et al. (2015). A NUCLEODUR^®^ 100-5 C18 column (150 × 4 mm ID) (5 μm) was used in conjunction with a ZORBAX Eclipse Plus C18 pre-column (4.6 × 12.5 mm) from Agilent.

Chromatographic analysis was performed with an elution gradient using a mobile phase of 5% acetic acid (solvent A) and acetonitrile (solvent B), for 0–35 min (0–92% B); 35 to 40 min (92–0% B); 40 to 42 min (0% B), for a total time of 42 min. The furnace was operated at 40°C. The injection volume was 20 μL, analyzed in triplicate, and the chromatographic acquisition was set at wavelengths in the 280 nm, 300 nm, and 320 nm regions (Table S3). Fifteen phenolic compounds were analyzed, of which analytical curves were constructed from dilutions of a 40 mg.L^−1^ stock solution containing all analytes dissolved in methanol. From the stock solution, dilutions were prepared for the construction of the analytical curves in a range of 0.5–15.0 mg.L^−1^. The limit of quantification ranged from 0.076 to 0.380 mg.L^−1^. The detection limit was in a range of 0.023 to 0.056 mg.L^−1^.

### 3.7. Statistical Analysis

The results were evaluated using ANOVA (one-way) analysis of variance and Tukey’s test to identify whether the changes in the parameters evaluated were significant at 95% confidence level. The principal component analysis (PCA) was performed to evaluate the influence of extraction methods with respect to the content of phenolic compounds (mgGAE·g^−1^), flavonoids (mgQE·g^−1^) and DPPH radical scavenging capacity (IC_50_, µg.mL^−1^), using the software PAST (Paleontological Statistics, Oslo, Norway) version 3.26. Since the averages for the aforementioned characterization tests use different units of measurement, the data were normalized in the range of 0 to 1.

## 4. Conclusions

The results showed that samples pretreated with medium temperature (50 °C) associated with ultrasound had higher antioxidant capacity (IC_50_ up to 33.86 ± 1.99 µg.mL^−1^) and total phenolic content (concentrations up to 439.05 ± 0.90 mgGAE·g^−1^). Based on the principal component analysis, it was observed that the samples obtained by SFE tended to have higher flavonoid contents (concentrations up to 50.47 ± 0.77 mgQE·g^−1^), an effect that was enhanced when this extraction technique was associated with sonication. In contrast, treatments with higher temperature (75 °C) showed lower total phenolic values (concentrations up to 23.38 ± 2.3 mgQE·g^−1^). In addition, by means of PCA, it was possible to observe that extracts obtained by SFE favored the obtainment of phenolic compounds such as rutin and kaempferol, with the SFE method being more selective in obtaining rutin (concentrations up to 10.00 ± 0.03 mg·g^−1^), an effect that was also strengthened with the use of sonication. Thus, it is hoped that these findings can support the elucidation of the use of pretreatment and type of extractive methods in the process of obtaining natural extracts composed of bioactive molecules.

## Figures and Tables

**Table 1:** Quantification of the nine major phenolic compounds by HPLC in different green propolis extracts obtained by ultrasound-assisted LPE and SFE.

Sample	Compounds (mg·g^−1^)								
	Quercetin	Gallic Acid	Formononetin	Kaempferol	*p*-Coumaric Acid	Caffeic Acid	Catechin	Epicatechin	Rutin
ST	2.30	0	13.37	1.17	14.03	1.21	1.05	0.51	1.93
A10	2.45	1.37	9.20	1.43	7.29	1.39	1.13	0.55	1.65
A20	2.37	0.68	6.04	1.01	7.53	1.69	1.37	0.98	0.21
A30	1.25	1.12	7.60	1.32	7.17	2.48	1.01	0.33	0.03
B10	1.01	1.48	14.80	0.88	14.33	1.48	0.71	0.22	2.71
B20	1.37	2.78	7.77	0.91	8.10	2.19	0.91	0.48	0.56
B30	2.24	0.44	6.66	0.78	0.00	3.02	1.16	11.49	0.48
C10	0.69	2.66	14.10	0.98	13.41	1.86	0.90	0.27	0.15
C20	0.67	2.62	14.31	0.93	12.56	1.76	0.83	0.39	1.42
C30	1.67	<LQ	11.90	0.89	12.20	2.06	0.93	0.66	1.75
ESC	2.06	2.15	8.39	0.34	7.87	0.23	0.66	0.51	4.17
UESC	0.68	1.79	4.76	2.51	0.00	0.19	0.52	0.31	14.99

**Table 2:** Pretreatment conditions for each green propolis extract obtained by ethanol (EtOH) (LPE) and supercritical (SCO_2_) extraction (SFE).

Sample	Treatment
ST	LPE Control
A10	25 °C, 10 min.
A20	25 °C, 20 min.
A30	25 °C, 30 min.
B10	50 °C, 10 min.
B20	50 °C, 20 min.
B30	50 °C, 30 min.
C10	75 °C, 10 min.
C20	75 °C, 20 min.
C30	75 °C, 30 min.
ESC	Control SFE
UESC	SFE, 50 °C, 20 min.

## Data Availability

Data are contained within the article.

## Supplementary Materials

The following supporting information can be downloaded at: https://www.mdpi.com/article/10.3390/molecules28052338/s1, Table S1: Antioxidant profile of green propolis extracts based on determination of total phenolic compounds, flavonoids, and antioxidant activity to DPPH (IC_50_); Figure S1: Graph of loadings for principal component 1; Figure S2: Graph of loadings for principal component 2; Table S2: Linear equations and their respective correlation coefficients used to calculate the IC_50_ of green propolis extracts; Table S3: HPLC-DAD data for the nine main quantified phenolic compounds: wavelength (λ), retention time (rt), concentration range (CR), limit of quantification (LQ) and limit of detection (LOD).

## Sample Availability

Samples of the compounds are available from the authors.
