# Supplementary material for: Extraction of Antioxidant Compounds from Brazilian Green Propolis Using Ultrasound-Assisted Associated with Low- and High-Pressure Extraction Methods
Source: Molecules. 2023 Mar 3;28(5):2338. doi: 10.3390/molecules28052338 (PMC10005562; doi:10.3390/molecules28052338)
Supplement: Supplementary file 1 [file molecules-28-02338-s001.zip › molecules-2235319-supplementary.pdf]

Supplementary Material

Table S1: Antioxidant profile of green propolis extracts based on determination of total phenolic compounds, flavonoids, and antioxidant activity to DPPH (IC_{50}).

Samples	Phenolic compounds (mgGAE.g^{-1})		Flavonoids (mgQE.g^{-1})		Antioxidant activity to DPPH (IC_{50}) (µg.mL^{-1})	
	Mean	SD	Mean	SD	Mean	SD
A10	342.00	14.87	29.14	3.98	113.93	7.07
A20	321.19	3.19	28.24	0.05	139.75	10.71
A30	335.53	7.88	27.76	1.52	124.65	16.23
B10	311.74	5.26	27.07	3.46	117.79	12.46
B20	439.05	0.90	24.84	0.28	33.86	0.25
B30	315.65	2.12	27.18	0.42	131.79	4.63
C10	349.37	2.88	23.38	2.30	112.37	1.15
C20	325.78	8.65	22.68	1.34	106.21	8.85
C30	386.29	12.63	18.82	1.15	108.85	6.27
ST	320.97	3.08	26.75	0.14	67.10	0.21
ESC	186.81	0.32	29.68	0.26	201.29	0.22
UESC	194.12	3.40	50.48	0.77	133.17	5.51

SD: standard deviations; GAE: gallic acid equivalents; QE: quercetin equivalents

Figure S1: Graph of loadings for principal component 1

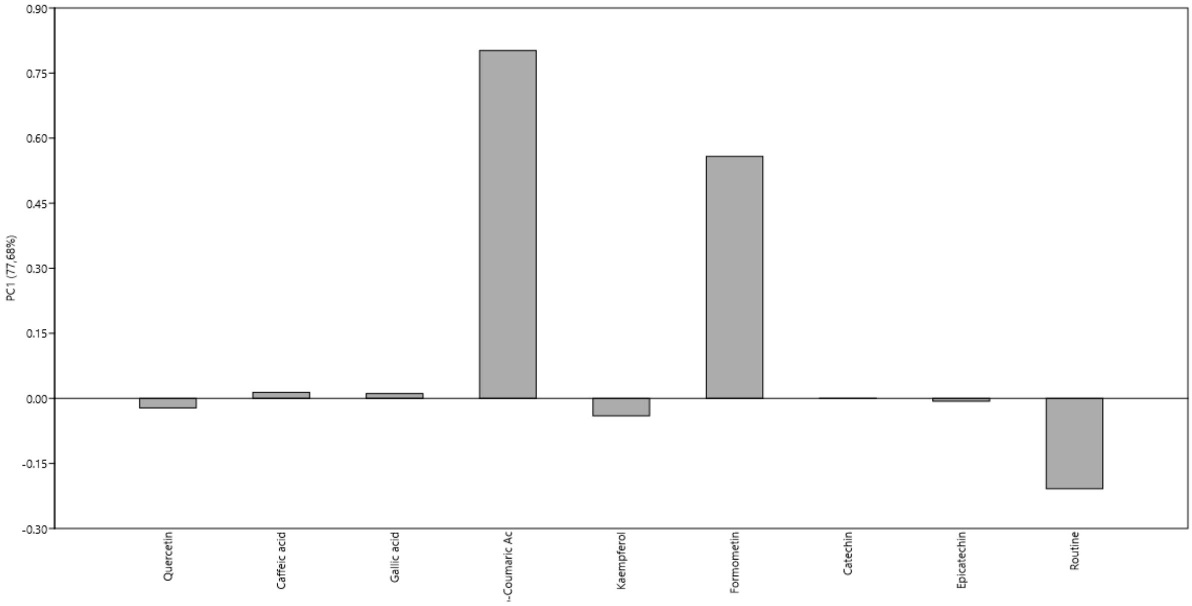

Figure S2: Graph of loadings for principal component 2.

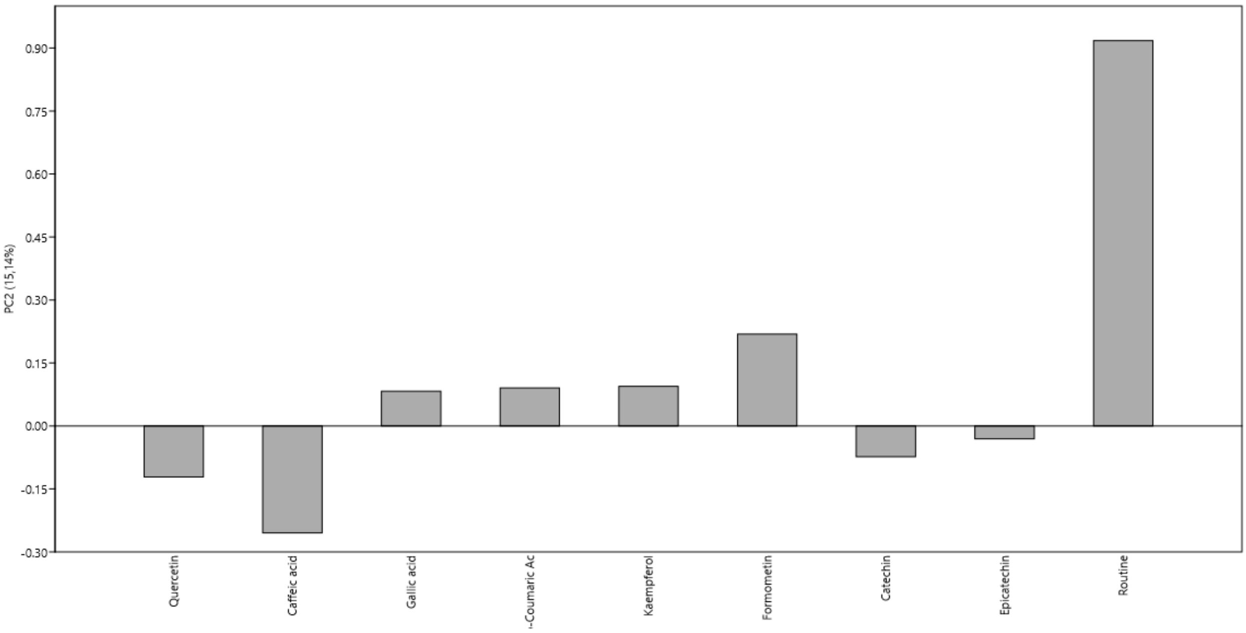

Table S2: Linear equations and their respective correlation coefficients used to calculate the IC_{50} of green propolis extracts.

Sample	Equation of the line	R^2
ST	y= 0,3194x + 4,3279	0,9918
A10	y= 0,3715x + 10,302	0,9992
A20	y= 0,3379x + 6,3991	0,9990
A30	y= 0,3235x + 4,4259	0,9994
B10	y= 0,4028 + 7,5728	0,9987
B20	y= 0,334x + 5,729	0,9923
B30	y= 0,03619x + 3,9787	0,9938
C10	y= 0,3805x + 7,6811	0,9916
C20	y= 0,4123x + 9,8587	0,9977
C30	y= 0,4344x +5,4372	0,9932
ESC	y= 0,2223x + 5,3016	0,9915
UESC	y= 0,3132x + 6,5645	0,9967

Table S3: HPLC-DAD data for the nine main quantified phenolic compounds: wavelength (λ), retention time (rt), concentration range (CR), limit of quantification (LQ) and limit of detection (LOD).

Standard	λ (nm)	rt (min.)	CR ($mg.L^{-1}$)	LQ ($mg.L^{-1}$)	LD ($mg.L^{-1}$)
Gallic Acid	280	2,315	0,5-15,0	0,187	0,056
Catechin	280	6,590	0,5-15,0	0,123	0,037
Epicatechin	280	8,449	0,5-15,0	0,080	0,024
Caffeic Acid	300	8,203	0,5-15,0	0,380	0,029
p-Coumaric Acid	300	10,353	0,5-15,0	0,078	0,023
Formononetin	300	19,275	0,5-15,0	0,076	0,023
Rutin	320	11,230	0,5-15,0	0,137	0,041
Quercetin	320	15,303	0,5-15,0	0,154	0,046
Kaempferol	320	17,501	0,5-15,0	0,097	0,029
